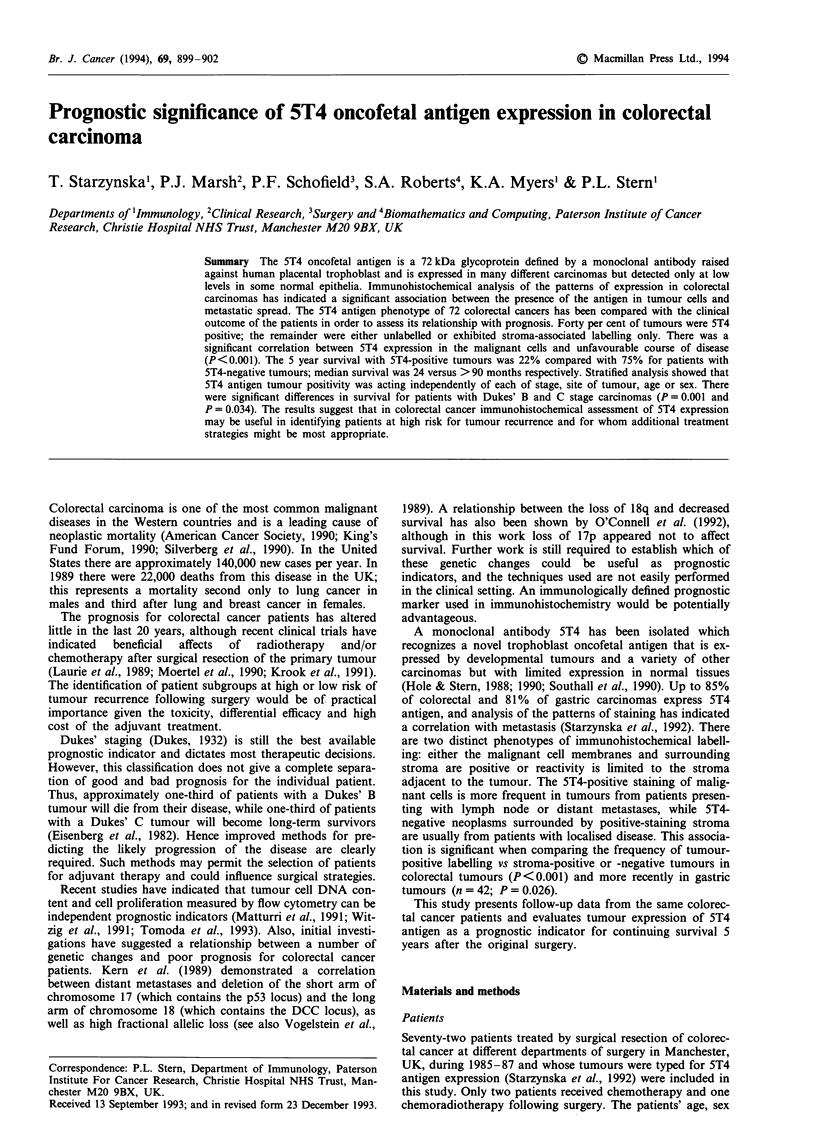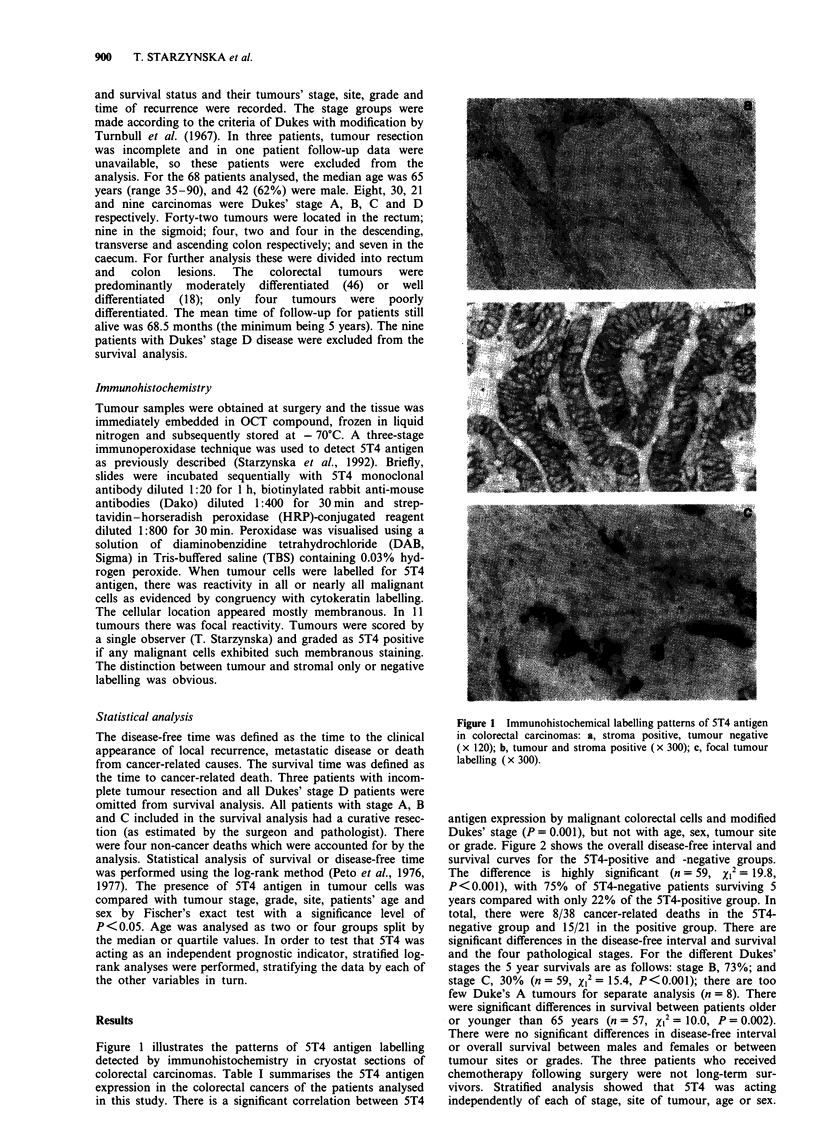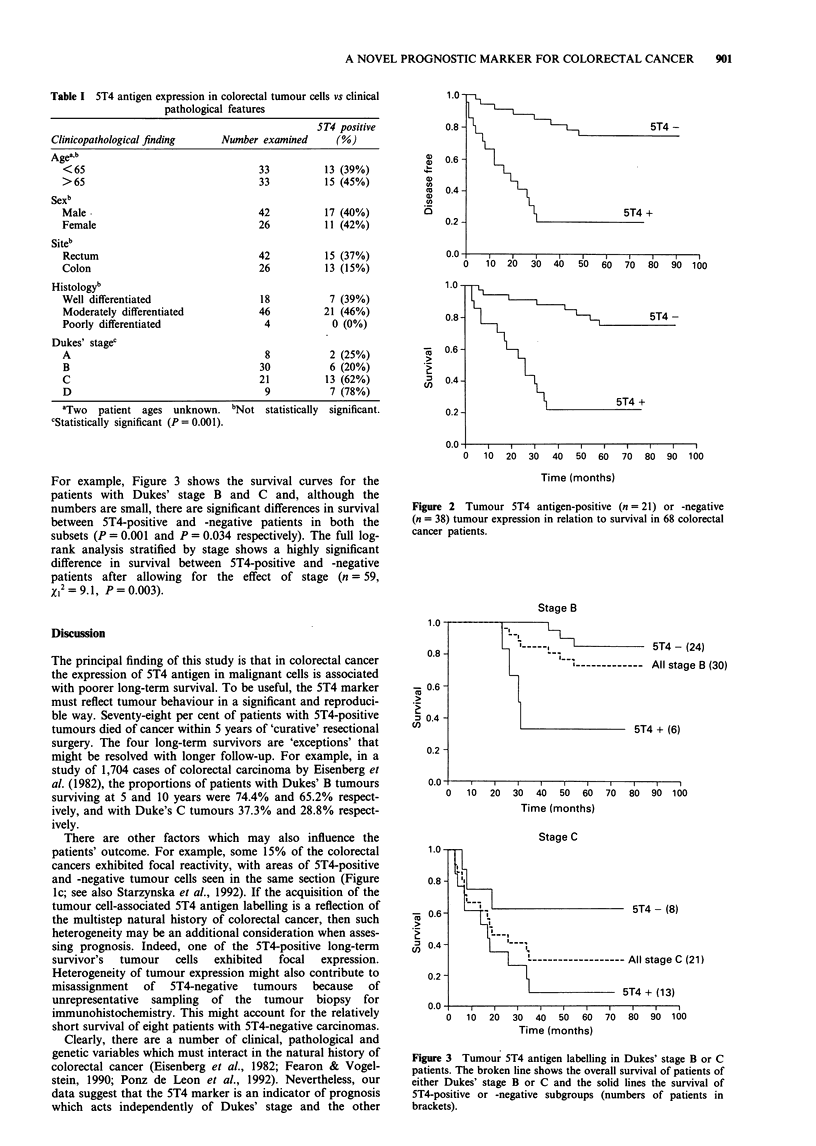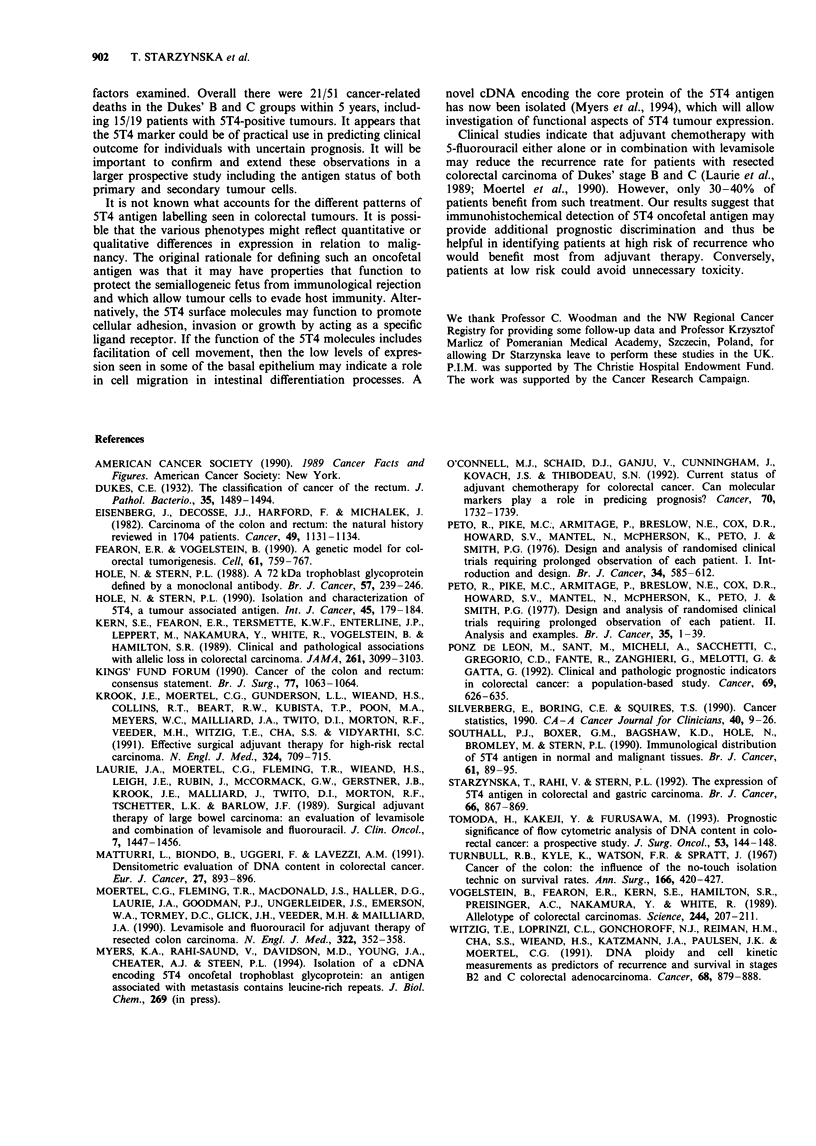# Prognostic significance of 5T4 oncofetal antigen expression in colorectal carcinoma.

**DOI:** 10.1038/bjc.1994.173

**Published:** 1994-05

**Authors:** T. Starzynska, P. J. Marsh, P. F. Schofield, S. A. Roberts, K. A. Myers, P. L. Stern

**Affiliations:** Department of Immunology, Paterson Institute of Cancer Research, Christie Hospital, NHS Trust, Manchester, UK.

## Abstract

**Images:**


Br. J. Cancer (1994), 69, 899-902 © Macmillan Press Ltd., 1994

### Prognostic significance of 5T4 oncofetal antigen expression in colorectal carcinoma

T. Starzynska[1], P.J. Marsh[2], P.F. Schofield[3], S.A. Roberts[4], K.A. Myers[1] & P.L. Stern[1]

*Departments of [1]Immunology, [2]Clinical Research, [3]Surgery and [4]Biomathematics and Computing, Paterson Institute of Cancer Research, Christie Hospital NHS Trust, Manchester M20 9BX, UK*

**Summary** The 5T4 oncofetal antigen is a 72 kDa glycoprotein defined by a monoclonal antibody raised against human placental trophoblast and is expressed in many different carcinomas but detected only at low levels in some normal epithelia. Immunohistochemical analysis of the patterns of expression in colorectal carcinomas has indicated a significant association between the presence of the antigen in tumour cells and metastatic spread. The 5T4 antigen phenotype of 72 colorectal cancers has been compared with the clinical outcome of the patients in order to assess its relationship with prognosis. Forty per cent of tumours were 5T4 positive; the remainder were either unlabelled or exhibited stroma-associated labelling only. There was a significant correlation between 5T4 expression in the malignant cells and unfavourable course of disease ($P < 0.001$). The 5 year survival with 5T4-positive tumours was 22% compared with 75% for patients with 5T4-negative tumours; median survival was 24 versus >90 months respectively. Stratified analysis showed that 5T4 antigen tumour positivity was acting independently of each of stage, site of tumour, age or sex. There were significant differences in survival for patients with Dukes' B and C stage carcinomas ($P = 0.001$ and $P = 0.034$). The results suggest that in colorectal cancer immunohistochemical assessment of 5T4 expression may be useful in identifying patients at high risk for tumour recurrence and for whom additional treatment strategies might be most appropriate.

Colorectal carcinoma is one of the most common malignant diseases in the Western countries and is a leading cause of neoplastic mortality (American Cancer Society, 1990; King's Fund Forum, 1990; Silverberg *et al.*, 1990). In the United States there are approximately 140,000 new cases per year. In 1989 there were 22,000 deaths from this disease in the UK; this represents a mortality second only to lung cancer in males and third after lung and breast cancer in females.

The prognosis for colorectal cancer patients has altered little in the last 20 years, although recent clinical trials have indicated beneficial affects of radiotherapy and/or chemotherapy after surgical resection of the primary tumour (Laurie *et al.*, 1989; Moertel *et al.*, 1990; Krook *et al.*, 1991). The identification of patient subgroups at high or low risk of tumour recurrence following surgery would be of practical importance given the toxicity, differential efficacy and high cost of the adjuvant treatment.

Dukes' staging (Dukes, 1932) is still the best available prognostic indicator and dictates most therapeutic decisions. However, this classification does not give a complete separation of good and bad prognosis for the individual patient. Thus, approximately one-third of patients with a Dukes' B tumour will die from their disease, while one-third of patients with a Dukes' C tumour will become long-term survivors (Eisenberg *et al.*, 1982). Hence improved methods for predicting the likely progression of the disease are clearly required. Such methods may permit the selection of patients for adjuvant therapy and could influence surgical strategies.

Recent studies have indicated that tumour cell DNA content and cell proliferation measured by flow cytometry can be independent prognostic indicators (Matturri *et al.*, 1991; Witzig *et al.*, 1991; Tomoda *et al.*, 1993). Also, initial investigations have suggested a relationship between a number of genetic changes and poor prognosis for colorectal cancer patients. Kern *et al.* (1989) demonstrated a correlation between distant metastases and deletion of the short arm of chromosome 17 (which contains the p53 locus) and the long arm of chromosome 18 (which contains the DCC locus), as well as high fractional allelic loss (see also Vogelstein *et al.*, 1989). A relationship between the loss of 18q and decreased survival has also been shown by O'Connell *et al.* (1992), although in this work loss of 17p appeared not to affect survival. Further work is still required to establish which of these genetic changes could be useful as prognostic indicators, and the techniques used are not easily performed in the clinical setting. An immunologically defined prognostic marker used in immunohistochemistry would be potentially advantageous.

A monoclonal antibody 5T4 has been isolated which recognizes a novel trophoblast oncofetal antigen that is expressed by developmental tumours and a variety of other carcinomas but with limited expression in normal tissues (Hole & Stern, 1988; 1990; Southall *et al.*, 1990). Up to 85% of colorectal and 81% of gastric carcinomas express 5T4 antigen, and analysis of the patterns of staining has indicated a correlation with metastasis (Starzynska *et al.*, 1992). There are two distinct phenotypes of immunohistochemical labelling: either the malignant cell membranes and surrounding stroma are positive or reactivity is limited to the stroma adjacent to the tumour. The 5T4-positive staining of malignant cells is more frequent in tumours from patients presenting with lymph node or distant metastases, while 5T4-negative neoplasms surrounded by positive-staining stroma are usually from patients with localised disease. This association is significant when comparing the frequency of tumour-positive labelling *vs* stroma-positive or -negative tumours in colorectal tumours ($P < 0.001$) and more recently in gastric tumours ($n = 42$; $P = 0.026$).

This study presents follow-up data from the same colorectal cancer patients and evaluates tumour expression of 5T4 antigen as a prognostic indicator for continuing survival 5 years after the original surgery.

### Materials and methods

### *Patients*

Seventy-two patients treated by surgical resection of colorectal cancer at different departments of surgery in Manchester, UK, during 1985–87 and whose tumours were typed for 5T4 antigen expression (Starzynska *et al.*, 1992) were included in this study. Only two patients received chemotherapy and one chemoradiotherapy following surgery. The patients' age, sex

Correspondence: P.L. Stern, Department of Immunology, Paterson Institute For Cancer Research, Christie Hospital NHS Trust, Manchester M20 9BX, UK.
Received 13 September 1993; and in revised form 23 December 1993.

and survival status and their tumours' stage, site, grade and time of recurrence were recorded. The stage groups were made according to the criteria of Dukes with modification by Turnbull et al. (1967). In three patients, tumour resection was incomplete and in one patient follow-up data were unavailable, so these patients were excluded from the analysis. For the 68 patients analysed, the median age was 65 years (range 35–90), and 42 (62%) were male. Eight, 30, 21 and nine carcinomas were Dukes' stage A, B, C and D respectively. Forty-two tumours were located in the rectum; nine in the sigmoid; four, two and four in the descending, transverse and ascending colon respectively; and seven in the caecum. For further analysis these were divided into rectum and colon lesions. The colorectal tumours were predominantly moderately differentiated (46) or well differentiated (18); only four tumours were poorly differentiated. The mean time of follow-up for patients still alive was 68.5 months (the minimum being 5 years). The nine patients with Dukes' stage D disease were excluded from the survival analysis.

*Immunohistochemistry*

Tumour samples were obtained at surgery and the tissue was immediately embedded in OCT compound, frozen in liquid nitrogen and subsequently stored at − 70°C. A three-stage immunoperoxidase technique was used to detect 5T4 antigen as previously described (Starzynska et al., 1992). Briefly, slides were incubated sequentially with 5T4 monoclonal antibody diluted 1:20 for 1 h, biotinylated rabbit anti-mouse antibodies (Dako) diluted 1:400 for 30 min and streptavidin–horseradish peroxidase (HRP)-conjugated reagent diluted 1:800 for 30 min. Peroxidase was visualised using a solution of diaminobenzidine tetrahydrochloride (DAB, Sigma) in Tris-buffered saline (TBS) containing 0.03% hydrogen peroxide. When tumour cells were labelled for 5T4 antigen, there was reactivity in all or nearly all malignant cells as evidenced by congruency with cytokeratin labelling. The cellular location appeared mostly membranous. In 11 tumours there was focal reactivity. Tumours were scored by a single observer (T. Starzynska) and graded as 5T4 positive if any malignant cells exhibited such membranous staining. The distinction between tumour and stromal only or negative labelling was obvious.

*Statistical analysis*

The disease-free time was defined as the time to the clinical appearance of local recurrence, metastatic disease or death from cancer-related causes. The survival time was defined as the time to cancer-related death. Three patients with incomplete tumour resection and all Dukes' stage D patients were omitted from survival analysis. All patients with stage A, B and C included in the survival analysis had a curative resection (as estimated by the surgeon and pathologist). There were four non-cancer deaths which were accounted for by the analysis. Statistical analysis of survival or disease-free time was performed using the log-rank method (Peto et al., 1976, 1977). The presence of 5T4 antigen in tumour cells was compared with tumour stage, grade, site, patients' age and sex by Fischer's exact test with a significance level of $P < 0.05$. Age was analysed as two or four groups split by the median or quartile values. In order to test that 5T4 was acting as an independent prognostic indicator, stratified log-rank analyses were performed, stratifying the data by each of the other variables in turn.

### Results

Figure 1 illustrates the patterns of 5T4 antigen labelling detected by immunohistochemistry in cryostat sections of colorectal carcinomas. Table I summarises the 5T4 antigen expression in the colorectal cancers of the patients analysed in this study. There is a significant correlation between 5T4 antigen expression by malignant colorectal cells and modified Dukes' stage ($P = 0.001$), but not with age, sex, tumour site or grade. Figure 2 shows the overall disease-free interval and survival curves for the 5T4-positive and -negative groups. The difference is highly significant ($n = 59$, $\chi_1^2 = 19.8$, $P < 0.001$), with 75% of 5T4-negative patients surviving 5 years compared with only 22% of the 5T4-positive group. In total, there were 8/38 cancer-related deaths in the 5T4-negative group and 15/21 in the positive group. There are significant differences in the disease-free interval and survival and the four pathological stages. For the different Dukes' stages the 5 year survivals are as follows: stage B, 73%; and stage C, 30% ($n = 59$, $\chi_1^2 = 15.4$, $P < 0.001$); there are too few Duke's A tumours for separate analysis ($n = 8$). There were significant differences in survival between patients older or younger than 65 years ($n = 57$, $\chi_1^2 = 10.0$, $P = 0.002$). There were no significant differences in disease-free interval or overall survival between males and females or between tumour sites or grades. The three patients who received chemotherapy following surgery were not long-term survivors. Stratified analysis showed that 5T4 was acting independently of each of stage, site of tumour, age or sex.

**Figure 1** Immunohistochemical labelling patterns of 5T4 antigen in colorectal carcinomas: **a**, stroma positive, tumour negative (× 120); **b**, tumour and stroma positive (× 300); **c**, focal tumour labelling (× 300).

**Table I** 5T4 antigen expression in colorectal tumour cells *vs* clinical pathological features

| *Clinicopathological finding* | *Number examined* | *5T4 positive (%)* |
|---|---|---|
| Age[a,b] | | |
| <65 | 33 | 13 (39%) |
| >65 | 33 | 15 (45%) |
| Sex[b] | | |
| Male | 42 | 17 (40%) |
| Female | 26 | 11 (42%) |
| Site[b] | | |
| Rectum | 42 | 15 (37%) |
| Colon | 26 | 13 (15%) |
| Histology[b] | | |
| Well differentiated | 18 | 7 (39%) |
| Moderately differentiated | 46 | 21 (46%) |
| Poorly differentiated | 4 | 0 (0%) |
| Dukes' stage[c] | | |
| A | 8 | 2 (25%) |
| B | 30 | 6 (20%) |
| C | 21 | 13 (62%) |
| D | 9 | 7 (78%) |

[a]Two patient ages unknown. [b]Not statistically significant. [c]Statistically significant ($P = 0.001$).

For example, Figure 3 shows the survival curves for the patients with Dukes' stage B and C and, although the numbers are small, there are significant differences in survival between 5T4-positive and -negative patients in both the subsets ($P = 0.001$ and $P = 0.034$ respectively). The full log-rank analysis stratified by stage shows a highly significant difference in survival between 5T4-positive and -negative patients after allowing for the effect of stage ($n = 59$, $\chi_1^2 = 9.1$, $P = 0.003$).

### Discussion

The principal finding of this study is that in colorectal cancer the expression of 5T4 antigen in malignant cells is associated with poorer long-term survival. To be useful, the 5T4 marker must reflect tumour behaviour in a significant and reproducible way. Seventy-eight per cent of patients with 5T4-positive tumours died of cancer within 5 years of 'curative' resectional surgery. The four long-term survivors are 'exceptions' that might be resolved with longer follow-up. For example, in a study of 1,704 cases of colorectal carcinoma by Eisenberg *et al.* (1982), the proportions of patients with Dukes' B tumours surviving at 5 and 10 years were 74.4% and 65.2% respectively, and with Duke's C tumours 37.3% and 28.8% respectively.

There are other factors which may also influence the patients' outcome. For example, some 15% of the colorectal cancers exhibited focal reactivity, with areas of 5T4-positive and -negative tumour cells seen in the same section (Figure 1c; see also Starzynska *et al.*, 1992). If the acquisition of the tumour cell-associated 5T4 antigen labelling is a reflection of the multistep natural history of colorectal cancer, then such heterogeneity may be an additional consideration when assessing prognosis. Indeed, one of the 5T4-positive long-term survivor's tumour cells exhibited focal expression. Heterogeneity of tumour expression might also contribute to misassignment of 5T4-negative tumours because of unrepresentative sampling of the tumour biopsy for immunohistochemistry. This might account for the relatively short survival of eight patients with 5T4-negative carcinomas.

Clearly, there are a number of clinical, pathological and genetic variables which must interact in the natural history of colorectal cancer (Eisenberg *et al.*, 1982; Fearon & Vogelstein, 1990; Ponz de Leon *et al.*, 1992). Nevertheless, our data suggest that the 5T4 marker is an indicator of prognosis which acts independently of Dukes' stage and the other

**Figure 2** Tumour 5T4 antigen-positive ($n = 21$) or -negative ($n = 38$) tumour expression in relation to survival in 68 colorectal cancer patients.

**Figure 3** Tumour 5T4 antigen labelling in Dukes' stage B or C patients. The broken line shows the overall survival of patients of either Dukes' stage B or C and the solid lines the survival of 5T4-positive or -negative subgroups (numbers of patients in brackets).

factors examined. Overall there were 21/51 cancer-related deaths in the Dukes' B and C groups within 5 years, including 15/19 patients with 5T4-positive tumours. It appears that the 5T4 marker could be of practical use in predicting clinical outcome for individuals with uncertain prognosis. It will be important to confirm and extend these observations in a larger prospective study including the antigen status of both primary and secondary tumour cells.

It is not known what accounts for the different patterns of 5T4 antigen labelling seen in colorectal tumours. It is possible that the various phenotypes might reflect quantitative or qualitative differences in expression in relation to malignancy. The original rationale for defining such an oncofetal antigen was that it may have properties that function to protect the semiallogeneic fetus from immunological rejection and which allow tumour cells to evade host immunity. Alternatively, the 5T4 surface molecules may function to promote cellular adhesion, invasion or growth by acting as a specific ligand receptor. If the function of the 5T4 molecules includes facilitation of cell movement, then the low levels of expression seen in some of the basal epithelium may indicate a role in cell migration in intestinal differentiation processes. A novel cDNA encoding the core protein of the 5T4 antigen has now been isolated (Myers et al., 1994), which will allow investigation of functional aspects of 5T4 tumour expression.

Clinical studies indicate that adjuvant chemotherapy with 5-fluorouracil either alone or in combination with levamisole may reduce the recurrence rate for patients with resected colorectal carcinoma of Dukes' stage B and C (Laurie et al., 1989; Moertel et al., 1990). However, only 30–40% of patients benefit from such treatment. Our results suggest that immunohistochemical detection of 5T4 oncofetal antigen may provide additional prognostic discrimination and thus be helpful in identifying patients at high risk of recurrence who would benefit most from adjuvant therapy. Conversely, patients at low risk could avoid unnecessary toxicity.

We thank Professor C. Woodman and the NW Regional Cancer Registry for providing some follow-up data and Professor Krzysztof Marlicz of Pomeranian Medical Academy, Szczecin, Poland, for allowing Dr Starzynska leave to perform these studies in the UK. P.I.M. was supported by The Christie Hospital Endowment Fund. The work was supported by the Cancer Research Campaign.

### References

AMERICAN CANCER SOCIETY (1990). *1989 Cancer Facts and Figures*. American Cancer Society: New York.

DUKES, C.E. (1932). The classification of cancer of the rectum. *J. Pathol. Bacterio.*, **35**, 1489–1494.

EISENBERG, J., DECOSSE, J.J., HARFORD, F. & MICHALEK, J. (1982). Carcinoma of the colon and rectum: the natural history reviewed in 1704 patients. *Cancer*, **49**, 1131–1134.

FEARON, E.R. & VOGELSTEIN, B. (1990). A genetic model for colorectal tumorigenesis. *Cell*, **61**, 759–767.

HOLE, N. & STERN, P.L. (1988). A 72 kDa trophoblast glycoprotein defined by a monoclonal antibody. *Br. J. Cancer*, **57**, 239–246.

HOLE, N. & STERN, P.L. (1990). Isolation and characterization of 5T4, a tumour associated antigen. *Int. J. Cancer*, **45**, 179–184.

KERN, S.E., FEARON, E.R., TERSMETTE, K.W.F., ENTERLINE, J.P., LEPPERT, M., NAKAMURA, Y., WHITE, R., VOGELSTEIN, B. & HAMILTON, S.R. (1989). Clinical and pathological associations with allelic loss in colorectal carcinoma. *JAMA*, **261**, 3099–3103.

KINGS' FUND FORUM (1990). Cancer of the colon and rectum: consensus statement. *Br. J. Surg.*, **77**, 1063–1064.

KROOK, J.E., MOERTEL, C.G., GUNDERSON, L.L., WIEAND, H.S., COLLINS, R.T., BEART, R.W., KUBISTA, T.P., POON, M.A., MEYERS, W.C., MAILLIARD, J.A., TWITO, D.I., MORTON, R.F., VEEDER, M.H., WITZIG, T.E., CHA, S.S. & VIDYARTHI, S.C. (1991). Effective surgical adjuvant therapy for high-risk rectal carcinoma. *N. Engl. J. Med.*, **324**, 709–715.

LAURIE, J.A., MOERTEL, C.G., FLEMING, T.R., WIEAND, H.S., LEIGH, J.E., RUBIN, J., MCCORMACK, G.W., GERSTNER, J.B., KROOK, J.E., MALLIARD, J., TWITO, D.I., MORTON, R.F., TSCHETTER, L.K. & BARLOW, J.F. (1989). Surgical adjuvant therapy of large bowel carcinoma: an evaluation of levamisole and combination of levamisole and fluorouracil. *J. Clin. Oncol.*, **7**, 1447–1456.

MATTURRI, L., BIONDO, B., UGGERI, F. & LAVEZZI, A.M. (1991). Densitometric evaluation of DNA content in colorectal cancer. *Eur. J. Cancer*, **27**, 893–896.

MOERTEL, C.G., FLEMING, T.R., MACDONALD, J.S., HALLER, D.G., LAURIE, J.A., GOODMAN, P.J., UNGERLEIDER, J.S., EMERSON, W.A., TORMEY, D.C., GLICK, J.H., VEEDER, M.H. & MAILLIARD, J.A. (1990). Levamisole and fluorouracil for adjuvant therapy of resected colon carcinoma. *N. Engl. J. Med.*, **322**, 352–358.

MYERS, K.A., RAHI-SAUND, V., DAVIDSON, M.D., YOUNG, J.A., CHEATER, A.J. & STEEN, P.L. (1994). Isolation of a cDNA encoding 5T4 oncofetal trophoblast glycoprotein: an antigen associated with metastasis contains leucine-rich repeats. *J. Biol. Chem.*, **269** (in press).

O'CONNELL, M.J., SCHAID, D.J., GANJU, V., CUNNINGHAM, J., KOVACH, J.S. & THIBODEAU, S.N. (1992). Current status of adjuvant chemotherapy for colorectal cancer. Can molecular markers play a role in predicing prognosis? *Cancer*, **70**, 1732–1739.

PETO, R., PIKE, M.C., ARMITAGE, P., BRESLOW, N.E., COX, D.R., HOWARD, S.V., MANTEL, N., MCPHERSON, K., PETO, J. & SMITH, P.G. (1976). Design and analysis of randomised clinical trials requiring prolonged observation of each patient. I. Introduction and design. *Br. J. Cancer*, **34**, 585–612.

PETO, R., PIKE, M.C., ARMITAGE, P., BRESLOW, N.E., COX, D.R., HOWARD, S.V., MANTEL, N., MCPHERSON, K., PETO, J. & SMITH, P.G. (1977). Design and analysis of randomised clinical trials requiring prolonged observation of each patient. II. Analysis and examples. *Br. J. Cancer*, **35**, 1–39.

PONZ DE LEON, M., SANT, M., MICHELI, A., SACCHETTI, C., GREGORIO, C.D., FANTE, R., ZANGHIERI, G., MELOTTI, G. & GATTA, G. (1992). Clinical and pathologic prognostic indicators in colorectal cancer: a population-based study. *Cancer*, **69**, 626–635.

SILVERBERG, E., BORING, C.E. & SQUIRES, T.S. (1990). Cancer statistics, 1990. *CA-A Cancer Journal for Clinicians*, **40**, 9–26.

SOUTHALL, P.J., BOXER, G.M., BAGSHAW, K.D., HOLE, N., BROMLEY, M. & STERN, P.L. (1990). Immunological distribution of 5T4 antigen in normal and malignant tissues. *Br. J. Cancer*, **61**, 89–95.

STARZYNSKA, T., RAHI, V. & STERN, P.L. (1992). The expression of 5T4 antigen in colorectal and gastric carcinoma. *Br. J. Cancer*, **66**, 867–869.

TOMODA, H., KAKEJI, Y. & FURUSAWA, M. (1993). Prognostic significance of flow cytometric analysis of DNA content in colorectal cancer: a prospective study. *J. Surg. Oncol.*, **53**, 144–148.

TURNBULL, R.B., KYLE, K., WATSON, F.R. & SPRATT, J. (1967) Cancer of the colon: the influence of the no-touch isolation technic on survival rates. *Ann. Surg.*, **166**, 420–427.

VOGELSTEIN, B., FEARON, E.R., KERN, S.E., HAMILTON, S.R., PREISINGER, A.C., NAKAMURA, Y. & WHITE, R. (1989). Allelotype of colorectal carcinomas. *Science*, **244**, 207–211.

WITZIG, T.E., LOPRINZI, C.L., GONCHOROFF, N.J., REIMAN, H.M., CHA, S.S., WIEAND, H.S., KATZMANN, J.A., PAULSEN, J.K. & MOERTEL, C.G. (1991). DNA ploidy and cell kinetic measurements as predictors of recurrence and survival in stages B2 and C colorectal adenocarcinoma. *Cancer*, **68**, 879–888.